# Conceptual growth curves of oropharyngeal soft tissues and craniofacial skeletal structures: clinical implications for pediatric airway management

**DOI:** 10.1007/s11325-026-03809-6

**Published:** 2026-09-19

**Authors:** Audrey J. Yoon, Jocelyn K. L. Hor, Kyung A. Kim

**Affiliations:** 1 Department of Orthodontics, Arthur A. Dugoni School of Dentistry, University of the Pacific, San Francisco, CA USA; 2 Division of Sleep Medicine, Department of Psychiatry and Behavioral Sciences, Stanford University School of Medicine, Redwood City, CA USA; 3 Department of Orthodontics, National Dental Centre Singapore, Singapore, Singapore; 4 Department of Orthodontics, College of Dentistry, Kyung Hee University, Seoul, Republic of Korea

**Keywords:** Oropharyngeal growth, Adenoids, Palatine tonsils, Lingual tonsils, Tongue, Obstructive sleep apnea, Scammon's growth curves, Craniofacial development, Pediatric airway, Sleep-disordered breathing

## Abstract

**Background:**

Pediatric obstructive sleep apnea (OSA) is closely linked to the growth and interaction of upper airway soft tissues. The relevance of Scammon’s classical growth curves to site-specific oropharyngeal development has been called into question, despite the fact that they have long provided a fundamental framework for comprehending lymphoid tissue maturation.

**Main body:**

This narrative review synthesizes current imaging-based and morphometric evidence on the age-related growth of the adenoids, palatine tonsils, lingual tonsils, and tongue, within the context of craniofacial development. Adenoidal tissue undergoes rapid early growth, peaking between 6 and 8 years — earlier than predicted by Scammon’s lymphoid curve. Palatine tonsils reach maximum dimensions between 8 and 10 years before gradual involution. Lingual tonsils lack normative developmental data in healthy children and appear largely absent on MRI in asymptomatic subjects, with hypertrophy representing a compensatory or condition-dependent response rather than normal development. The tongue follows a general-type growth trajectory with marked regional differences: the anterior two-thirds attains near-adult size by 8–10 years, while the posterior third continues enlarging into adolescence. These structure-specific trajectories interact with the differential maturation of craniofacial structures — the cranium, maxilla, and mandible — creating a transient “window of vulnerability” between approximately 5 and 10 years of age, during which soft tissue volume disproportionately narrows the upper airway.

**Conclusions:**

Oropharyngeal soft tissue growth is structure-specific and does not conform to a single generalized growth curve. A conceptual, site-specific clinical framework integrating the distinct growth trajectories of each tissue and their proportional relationship to craniofacial development is essential for orthodontic assessment, airway risk stratification, and timely intervention in children with or at risk for sleep-disordered breathing.

## Introduction

Pediatric obstructive sleep apnea (OSA) is a prevalent respiratory condition in children, characterized by recurrent upper airway obstruction during sleep [1, 2]. Its etiology is multifactorial and reflects an imbalance between factors that maintain airway patency and those that promote airway collapse. In children, this imbalance is closely related to the development and interaction of upper airway soft tissues. Enlargement of oropharyngeal lymphoid tissues, particularly adenotonsillar hypertrophy, is the most common anatomical factor in pediatric OSA and may compromise airway patency not only by absolute size but also by its proportion relative to the airway dimension [3, 4]. The tongue is another key soft tissue component of the upper airway. Accordingly, the growth of upper airway soft tissues including the adenoids, palatine tonsils, lingual tonsils, and tongue collectively influences the spatial relationship between soft tissue volume and the airway dimension within the craniofacial skeletal framework, highlighting the critical importance of understanding age-related growth and developmental patterns of oropharyngeal soft tissues.

The principles of human growth and development have been classically described by Richard E. Scammon, who introduced distinct growth curves for neural, general, lymphoid, and genital tissues in his landmark work *The Measurement of the Body in Childhood* (1930) [5]. These growth patterns provide a foundational framework for understanding age-related physiological changes, distinguishing normal from abnormal development, and determining the timing and necessity of clinical intervention. However, as this review highlights, site-specific evidence has more recently cast doubt on the applicability of Scammon’s classical description of human growth patterns to oropharyngeal tissues, whose development appears more complex and heterogeneous than classical models imply. A narrative review by Hosomichi and Ono emphasized that the growth patterns of oropharyngeal lymphoid tissues are more heterogeneous and dynamic than suggested by classical models, underscoring the need for a more nuanced and integrative approach in clinical interpretation [6].

In this context, this narrative review aims to provide updated insights into the growth of oropharyngeal soft tissues — including the adenoids, palatine tonsils, lingual tonsils, and tongue — by integrating current evidence within a conceptual framework of growth trajectories, to better reflect the complex and dynamic nature of upper airway soft tissue development.

## Literature search and selection criteria

The methodology for this narrative review was structured in alignment with the Scale for the Assessment of Narrative Review Articles (SANRA) framework [7]. A targeted literature search was conducted utilizing three primary electronic medical databases: PubMed/MEDLINE, Scopus, and the Web of Science Core Collection.

The search strategy was constructed using a combination of Medical Subject Headings (MeSH) and free-text keywords to identify relevant anatomical and clinical studies. The primary search strings included anatomical and developmental terms such as “growth”, “tonsils”, “palatine”, “adenoids”, “volume”, “size”, “tongue”, and “healthy children”. These terms were subsequently combined using Boolean operators with the primary target clinical concepts: “obstructive sleep apnea”, “sleep-disordered breathing”, and “adenotonsillar hypertrophy”.

Article selection was governed by predefined inclusion and exclusion criteria to capture high-quality, relevant data while mitigating selection bias. Inclusion criteria were restricted to peer-reviewed clinical studies, longitudinal and cross-sectional morphometric analyses, three-dimensional volumetric imaging studies (including magnetic resonance imaging and cone-beam computed tomography), and outcome studies evaluating clinical or orthodontic interventions in pediatric cohorts. Foundational theoretical papers concerning classical craniofacial and soft tissue growth trajectories were also included to provide necessary conceptual context. Conversely, the exclusion criteria removed non-peer-reviewed commentaries, editorials, single case reports, and studies lacking objective quantitative structural, volumetric, or polysomnographic data regarding upper airway development. Only articles published in the English language were considered for this review.

## A conceptual perspective on scammon’s growth curves

In the early 20th century, Scammon analyzed extensive anthropometric and anatomic datasets including post-mortem measurements and organ weights into a set of growth curves describing four major tissue systems: general, neural, genital, and lymphoid [5]. By expressing organ size as a proportion of adult values, this framework demonstrated that tissues exhibit distinct growth velocities and maturational timing. This principle has since been extended to craniofacial biology: Proffit adapted Scammon’s framework to illustrate differential growth patterns of the maxilla and mandible, including evidence of both a childhood growth spurt (approximately 7.5 years) and an adolescent peak (approximately 12.7 years) in mandibular development [8]. These observations reinforced the concept that tissue-specific growth trajectories underlie normal and aberrant craniofacial development.

**Fig. 1:**
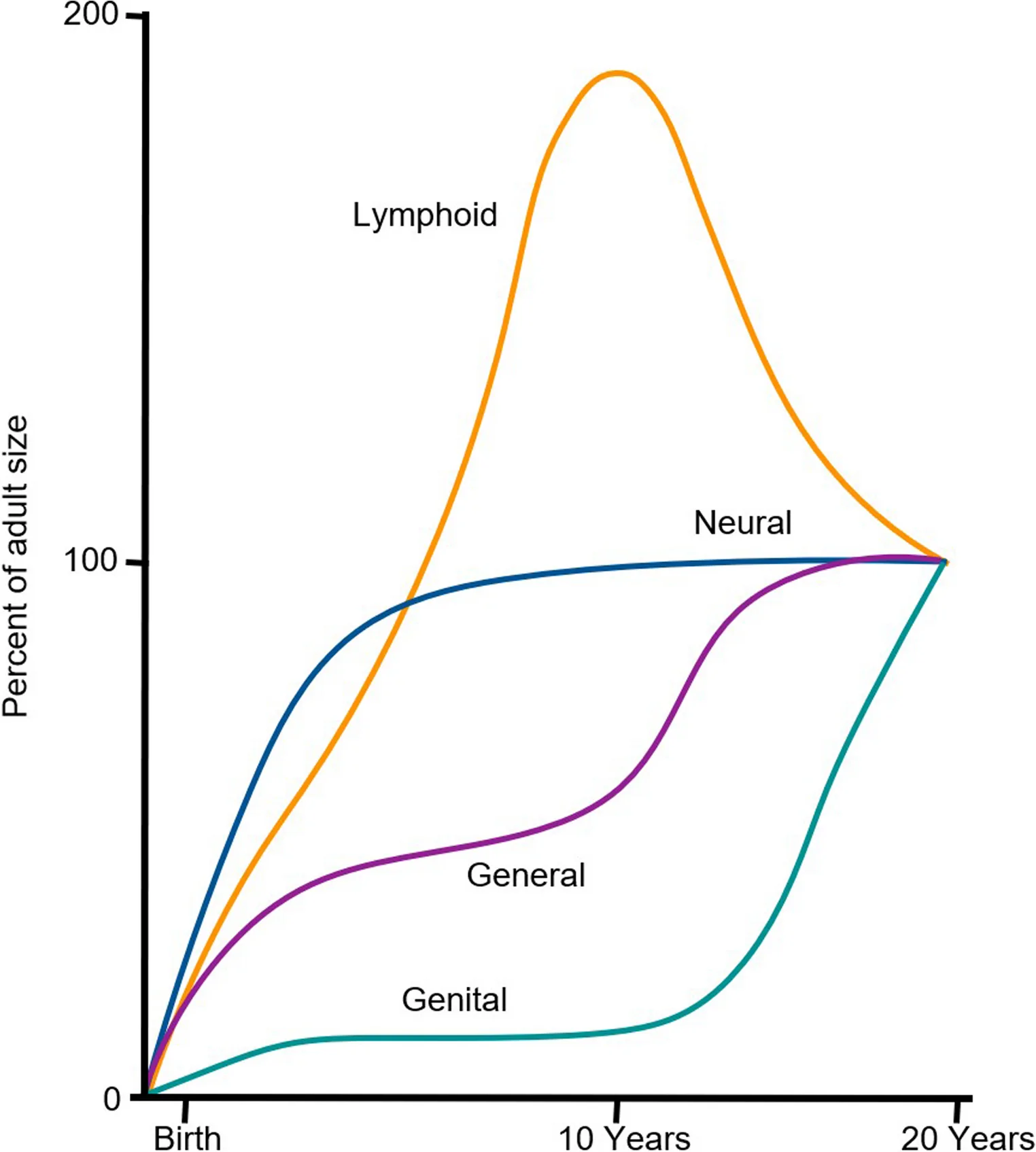
Scammon’s four classical tissue growth curves (neural, general, genital, and lymphoid) The lymphoid curve — derived primarily from thymic growth data — is shown reaching approximately 200% of adult size by early adolescence before declining. The thymic trajectory is used to illustrate the degree to which pharyngeal lymphoid tissues (adenoids, palatine tonsils) deviate from this classical pattern, with site-specific growth trajectories superimposed to highlight earlier peak timing and less pronounced overshoot

Lymphoid tissues undergo rapid proliferation during childhood as part of immune system maturation, providing protection against frequent early-life infections. The thymus, the primary lymphoid organ located in the anterior mediastinum, supports T-lymphocyte maturation and shows substantial growth in early life, reaching peak size around puberty before undergoing involution, during which functional parenchyma is progressively replaced by adipose and connective tissue. This developmental trajectory is reflected in Scammon’s lymphoid growth curve, which demonstrates rapid expansion to approximately 200% of adult size, peaking around 12 years of age before declining (Fig. 1) [5]. In contrast, the adenoids and palatine tonsils, which constitute key components of Waldeyer’s ring in the pharynx, function as the first line of defense against inhaled and ingested pathogens. Although these tissues play a critical immunological role during early childhood, their functional importance diminishes with age as other immune mechanisms mature.

Given its role as the primary lymphoid organ, the lymphoid growth curve proposed by Scammon was derived largely from thymic development and was assumed to represent other lymphoid structures, such as the adenoids, palatine tonsils, and Peyer’s patches. However, recent evidence suggests that these tissues do not follow a uniform developmental trajectory. Imaging-based studies have demonstrated variability in both the timing and magnitude of pharyngeal lymphoid tissue growth, indicating that individual structures exhibit distinct and non-synchronous patterns [9–11]. Further support is provided by longitudinal observations during the preadolescent period (approximately 8–12 years), in which palatine tonsil size tends to remain relatively stable, whereas adenoidal tissue continues to increase [12]. These findings underscore heterogeneity in lymphoid tissue growth that cannot be fully captured by a single generalized growth curve.

In addition to lymphoid tissues, the tongue is a critical component of upper airway anatomy whose growth pattern is not explicitly defined in Scammon’s original classification. As a muscle-derived organ, the tongue is generally considered to follow a general-type growth trajectory characterized by progressive enlargement with somatic growth [13]. Its development is also closely linked to surrounding craniofacial structures. Morphometric and imaging studies show that tongue size and position are related to the mandible, hyoid bone, and pharyngeal airway, indicating coordinated rather than independent growth. Consistent with Moss’s Functional Matrix Theory, changes in tongue size, posture, or function can influence craniofacial morphology and airway dimensions [14].

Altered tongue posture has been associated with maxillary constriction and transverse arch deficiency [15]. Reduced tongue muscle tone may contribute to the development of malocclusion and Class II skeletal patterns [16]. Restricted tongue mobility — as seen in ankyloglossia — has been identified as a functional risk factor for maxillary hypoplasia and soft palate elongation [17]. In contrast, increased tongue volume [18] or posterior tongue base displacement [19] may reduce retroglossal airway dimensions, and tonsillar hypertrophy combined with posterior tongue positioning has been associated with increased susceptibility to sleep-disordered breathing in children [20]. Taken together, these observations suggest that upper airway soft tissue development is better characterized by structure-specific growth patterns and their proportional relationship to the surrounding craniofacial framework, rather than by a single uniform trajectory. This interpretation represents an integration of the reviewed evidence rather than a finding directly demonstrated by any single study.

## Oropharyngeal soft tissue growth and craniofacial development

To characterize these growth patterns, imaging modalities such as magnetic resonance imaging (MRI), ultrasonography (US), computed tomography (CT), and cephalometric radiography have been widely utilized. These studies consistently demonstrate rapid early growth of lymphoid tissues, likely associated with increased antigenic exposure in childhood, followed by variable patterns of change depending on age and functional demand. In addition, differences in growth patterns have been observed between healthy children and those with OSA.

### Adenoids

The adenoid is located on the posterosuperior wall of the nasopharynx and represents a major component of the upper airway during childhood. Early lateral radiographic studies demonstrated that the adenoids are observable as early as 6 weeks of age, undergo rapid enlargement by approximately 4 years, reach peak size around 12 years, and subsequently regress during adolescence, becoming minimal or absent by early adulthood [21]. Handelman and Osborne further reported that adenoid growth and involution generally conform to Scammon’s lymphoid growth curve, particularly in the absence of recurrent adenitis [22].

However, Pruzansky reported that adenoid growth does not follow a fixed, uniform trajectory but instead reflects individual responses to varying physiological and environmental influences [23]. Longitudinal observations have demonstrated that adenoid growth deviates from this classical pattern in both timing and magnitude. Linder-Aronson et al. reported that adenoid size does not show a continuous increase toward puberty and may exhibit minor secondary increases around 10 years of age, possibly associated with pubertal hormonal influences [24]. MRI-based studies have shown that the adenoid pad can be detected as early as 4–5 months of age, with rapid enlargement occurring primarily between 3 and 5 years [13, 25, 26]. This early phase of growth is associated with increased antigenic stimulation and a reduction in nasopharyngeal airway space. Thereafter, growth proceeds at a slower rate, with peak size typically observed earlier than previously reported — most commonly between 7 and 8 years of age rather than during adolescence [11]. Consistent with these findings, Vogler et al. demonstrated relative stability of adenoid size after early childhood, followed by gradual regression [9].

Ishida et al. analyzed adenoid size from lateral cephalometric radiographs in Japanese individuals aged 6–20 years and found no consistent pattern of decline across age groups [27] A significant reduction in adenoid size was observed only when comparing younger children (mean age 8.21 ± 0.96 years) with young adults (mean age 18.86 ± 1.08 years). Analyses have further demonstrated that adenoid growth varies according to the specific dimension measured. Adenoid depth, measured along the maxillary plane, reaches its maximum at approximately 4 years of age, whereas height and thickness peak later, around 7–8 years [10]. Involution occurs sequentially, beginning with a reduction in depth followed by decreases in height and thickness [10]. These findings indicate that adenoid growth is not a single uniform process but varies depending on the specific dimension measured. Although direct comparison with Scammon’s model is limited due to methodological differences, these findings suggest that adenoid growth does not follow the uniform enlargement and involution pattern described in the classical lymphoid-type curve.

### Palatine tonsils

Similarly, the growth pattern of the palatine tonsils demonstrates distinct age-dependent characteristics that are not fully aligned with Scammon’s classical lymphoid growth curve [5]. The palatine tonsils, paired lymphoid structures located in the oropharynx between the palatoglossal and palatopharyngeal arches, have traditionally been evaluated using the Brodsky grading system based on oropharyngeal inspection. Clinical observations indicate that younger children (≤ 7 years) exhibit significantly larger tonsils than older children (> 7 years), suggesting that tonsillar prominence is greatest in early childhood and declines thereafter [28]. Ultrasonographic imaging has demonstrated that tonsillar volume increases rapidly in early life, with significant growth occurring up to approximately 3 years of age, followed by minimal change between 3 and 12 years [29, 30]. In a large cohort of asymptomatic children, Hosokawa et al. [29] reported early volume expansion without subsequent enlargement beyond early childhood, whereas Aydin et al. [30] demonstrated a mean tonsil volume of approximately 1.5 ± 0.9 cm³, with positive correlations with somatic growth parameters but no differences by sex or laterality.

MRI-based evaluation provides additional insight into absolute size and its relationship to airway development. Papaioannou et al. [11] demonstrated that palatine tonsils increase during early childhood and reach maximal dimensions between 8 and 10 years of age, depending on the measurement method, followed by progressive reduction thereafter. Notably, the oropharyngeal airway shows minimal expansion before approximately 8 years of age and enlarges thereafter, resulting in a progressive decrease in the relative contribution of tonsillar tissue to airway narrowing with growth. By adulthood, tonsillar tissue is markedly reduced, persisting only as small lymphoid remnants within Waldeyer’s ring [27].

**Fig. 2:**
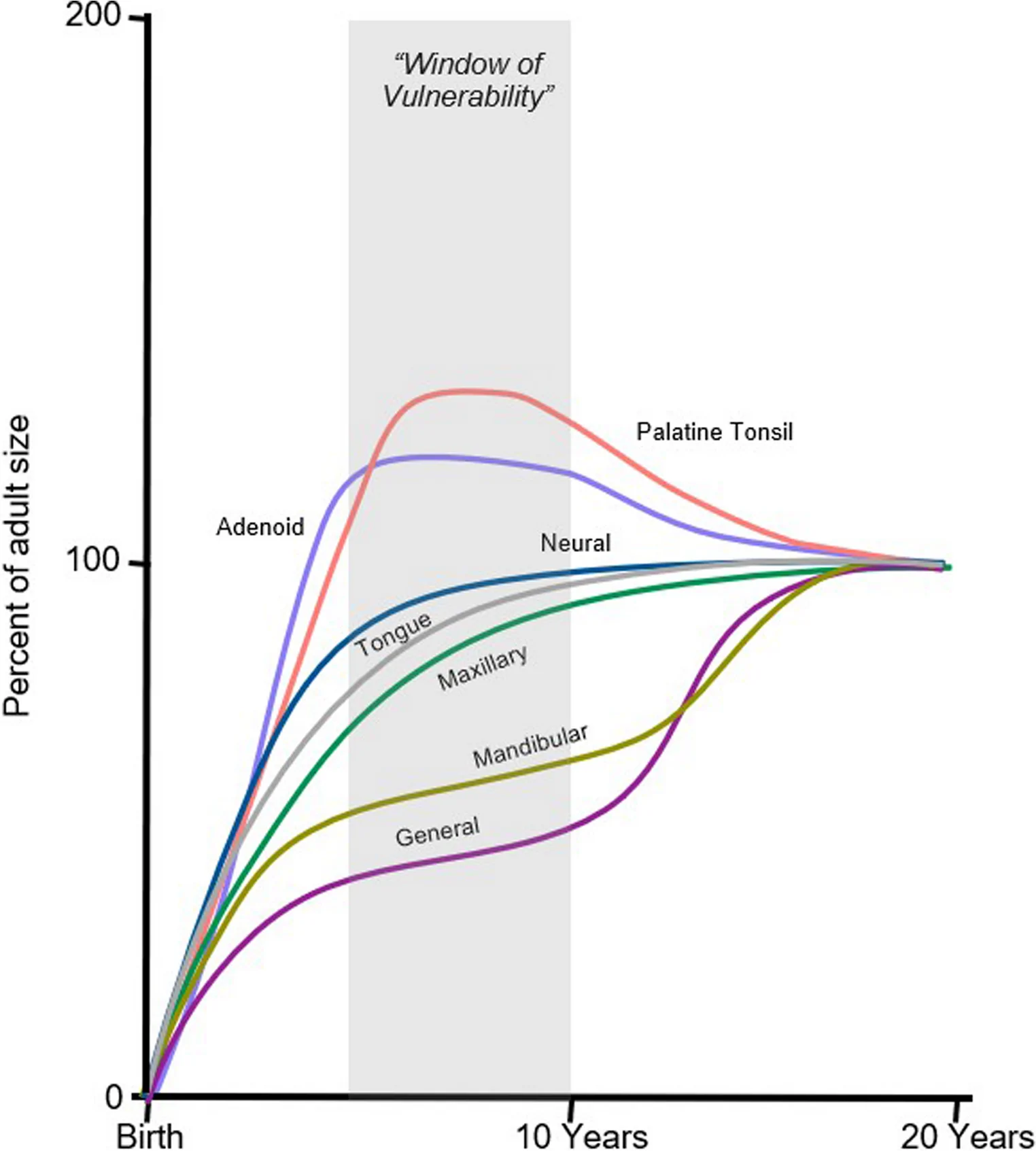
An updated interpretation of Scammon’s growth curves that incorporates site-specific deviations observed in pharyngeal lymphoid tissues (adenoids and palatine tonsils), characterized by earlier attainment of maximal size, a sustained plateau through early childhood, and modest overshoot (approximately 125% of adult size for the adenoid and 145% for the palatine tonsil, derived from Ishida et al. [27]) rather than the ~ 200% predicted by the classical lymphoid curve. Conceptual schematic illustration of the temporal growth trajectories of major oropharyngeal soft tissues — adenoids, palatine tonsils, lingual tonsils, and tongue — relative to the maturation of craniofacial skeletal structures (cranium, maxilla, mandible). The “window of vulnerability” (approximately 5–10 years of age) is indicated, during which peak lymphoid tissue volume coincides with incomplete craniofacial skeletal development, resulting in maximal relative airway compromise

Taken together, these findings indicate that palatine tonsil growth is characterized by early enlargement, a mid-childhood peak (approximately 8–10 years), and subsequent gradual involution. This trajectory differs from Scammon’s lymphoid growth curve, which predicts continued enlargement until preadolescence with more pronounced overshoot relative to adult size. Compared with adenoidal tissue, palatine tonsils demonstrate a slightly later peak and a less pronounced early airway impact, reflecting differences in both growth timing and spatial relationship to the airway [27] (Fig. 2).

### Lingual tonsils

**Fig. 3:**
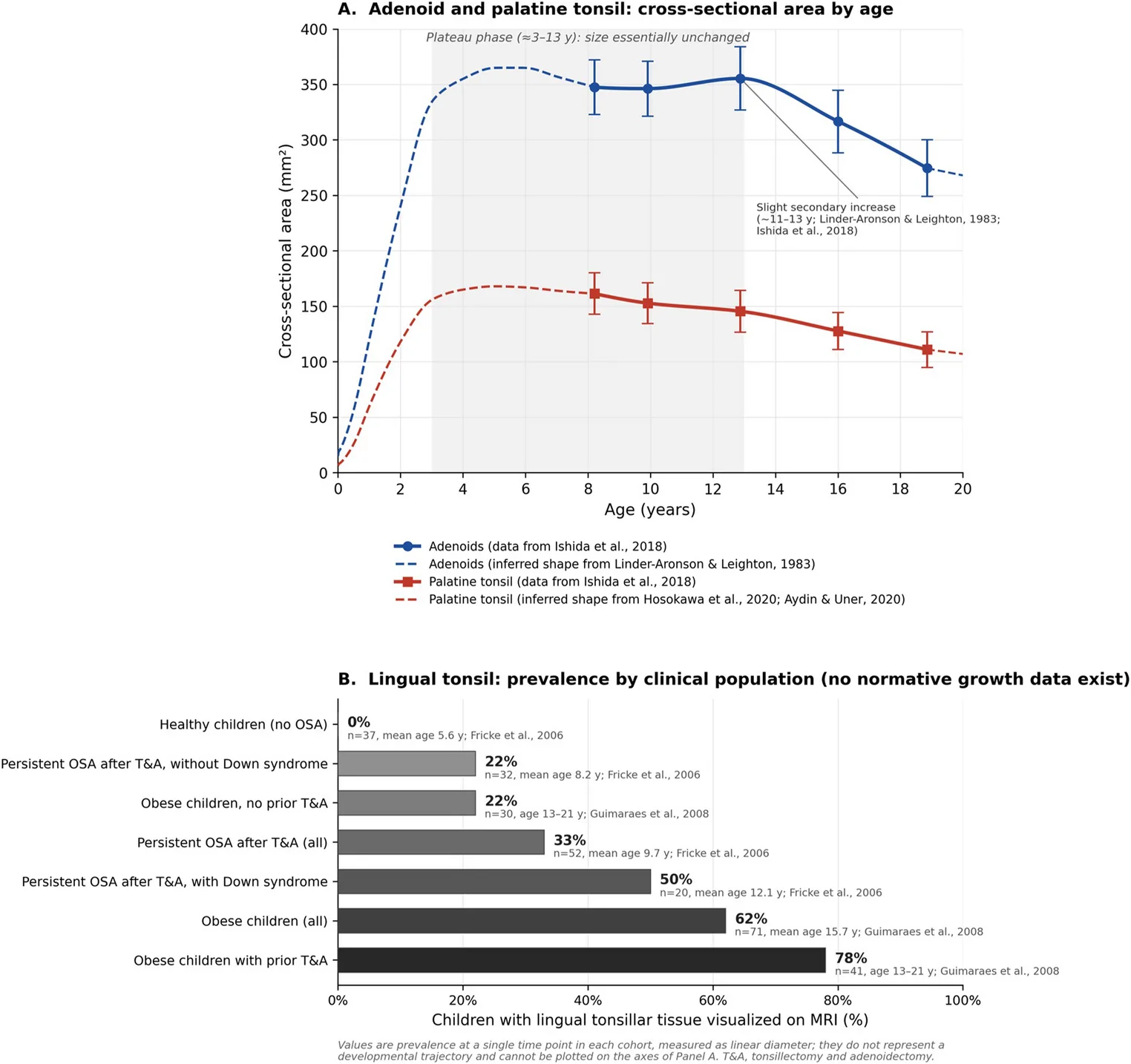
Growth trajectories of the adenoid and palatine tonsil, and prevalence of lingual tonsil enlargement. (**A**) Cross-sectional area (mm²) of the adenoid and palatine tonsil from birth to 20 years. Solid lines with error bars (mean ± 95% CI) denote measured data adapted from Ishida et al. [27], a longitudinal cephalometric cohort (*n* = 90) in which adenoid and palatine tonsil area were measured in the same subjects at 8.21, 9.91, 12.88, 16.01, and 18.86 years using a validated method. In that cohort, neither adenoid nor palatine tonsil area differed significantly between any consecutive age groups; a significant reduction was detected only between the youngest (8.21 y) and oldest (18.86 y) groups (*p* < 0.01). Dashed lines denote an inferred trajectory shape, not measured area data, as no cross-sectional area measurements exist for this age range in the literature reviewed. For the adenoid, the dashed segment follows the pattern described by Linder-Aronson and Leighton [24], whose longitudinal cephalometric study (*n* = 56, ages 3–16 years) found posterior nasopharyngeal soft-tissue thickness to be already near-maximal at 3 years, largest at 5–6 years, and only marginally smaller at 8 years, indicating a sustained plateau through early childhood rather than a discrete peak; the slight secondary increase at 10–11 years reported in that study corresponds to the modest rise at 12.88 years in the Ishida cohort. For the palatine tonsil, the dashed segment reflects the rapid early volumetric expansion to approximately 3 years followed by minimal change reported by Hosokawa et al. [29] and Aydin and Uner [30]. Because these sources used linear or volumetric measures rather than cross-sectional area, the dashed portions are illustrative of overall shape only and are not directly comparable to the solid portions. The shaded band indicates the plateau phase (approximately 3–13 years) during which absolute tissue size is essentially unchanged. (**B**) Prevalence of lingual tonsillar tissue visualized on MRI across clinical populations. No lingual tonsil tissue was perceptible in asymptomatic children without OSA [32]. Prevalence rises progressively with persistent OSA after adenotonsillectomy, Down syndrome, and obesity, reaching 78% in obese children with prior adenotonsillectomy [31]. Because these are single-time-point prevalence data from non–age-matched cohorts, measured as linear diameter rather than cross-sectional area, they cannot support a developmental trajectory and are therefore presented separately from Panel A. T&A, tonsillectomy and adenoidectomy; OSA, obstructive sleep apnea

The lingual tonsils, located at the base of the tongue, exhibit a growth pattern distinct from the adenoids and palatine tonsils. Unlike the adenoids and palatine tonsils, lingual tonsillar tissue is not perceptible on MRI in asymptomatic children and has been documented almost exclusively in clinical populations (Fig. 3B). Guimaraes et al. reported that forty-four (62%) of the obese children had measurable lingual tonsils, which is greater than the frequency previously reported in normal subjects (0%), subjects with obstructive sleep apnea (33%), or subjects with Down syndrome and obstructive sleep apnea (50%) and these observations may suggest that lingual tonsil hypertrophy may contribute to the pathogenesis of obstructive sleep apnea in obese children [31]. While MRI, CT, and endoscopic studies have been used to evaluate lingual tonsils, these investigations have predominantly involved children with sleep-disordered breathing, obesity, syndromic conditions, or other risk factors, and employ heterogeneous assessment methods without standardized measurement criteria. There is a significant gap in literature, as there is currently no established normative growth trajectory for lingual tonsils in healthy children, likely due to the limited visibility of these structures with routine, non-endoscopic imaging techniques. Fricke et al. [32] reported that lingual tonsillar tissue was not visualized on MRI in asymptomatic children without OSA, whereas it was present in 33% of children with persistent OSA following adenotonsillectomy, with a mean diameter of 9.50 ± 4.6 mm and marked enlargement (> 10 mm) in 15% of cases. Lingual tonsil enlargement has also been reported more frequently in children with persistent OSA and in those with Down syndrome [32–34]. In this context, lingual tonsillar hypertrophy is considered a condition-dependent or compensatory response — particularly following removal of other lymphoid tissues — rather than a predictable component of normal lymphoid development.

### Tongue

Among oropharyngeal soft tissue structures, the tongue represents a key component requiring detailed evaluation. Beyond its role in airway patency and collapsibility, tongue size, posture, and function influence craniofacial development, as described by Moss’ Functional Matrix Theory [14].

The tongue is a muscular organ occupying the oral cavity, with the tongue base forming the anterior wall of the oropharynx. Unlike adenoids and palatine tonsils, the tongue is a muscle-derived structure, and its development has been investigated using morphometric and regional growth analyses. In a cross-sectional morphometric study, Siebert [35] examined 83 normal tongues from 25 weeks of gestation to 10.5 years of age, measuring weight, length, width, thickness, and volume in relation to somatic and craniofacial parameters. From fetal life through childhood, tongue dimensions increase progressively without lymphoid-type overshoot. Linear dimensions increase by approximately 1.5-fold from birth to 10.5 years, whereas tongue weight increases substantially — reaching approximately tenfold of birth values [35]. Tongue weight correlates strongly with body length and body weight, whereas tongue length shows the strongest correlation with head circumference, accounting for 85.4% of variance [35].

Tongue growth shows clear regional differences. The anterior two-thirds reaches approximately 70% of adult size by 6 years and attains near-adult dimensions by 8–10 years, whereas the posterior third continues to grow until 15–16 years [36, 37]. Direct measurements in 232 subjects aged 4–32 years demonstrate minimal change in anterior midline length after mid-childhood (15.7 mm at 4–7 years; 18.3 mm at 8–10 years; 17.4 mm in adults), while posterior midline length increases continuously (32.2 mm to 40.6 mm to 50.4 mm). Anterior tongue area increases from 674 mm² in early childhood to 1,027 mm² in adulthood, whereas posterior tongue area increases more markedly from 901 mm² to 1,816 mm².

These findings indicate that tongue development resembles a general-type growth pattern with continuous enlargement. However, growth is not uniform: anterior regions mature earlier, whereas posterior regions continue to expand during adolescence, reflecting strong dependence on craniofacial development.

### Craniofacial growth

The region-specific and craniofacially dependent growth pattern of the tongue is consistent with the differential maturation of the craniofacial complex. Contemporary longitudinal data from Buschang and colleagues further clarify this relationship, demonstrating a postnatal maturity gradient in which craniofacial structures are distributed between neural and general growth trajectories [38–40].

Within this framework, the cranium follows a neural pattern and reaches approximately 94% of adult size by 6 years of age, whereas the midface and mandible remain less mature and follow growth patterns more consistent with general somatic development [38–40]. Maxillary growth shows its highest velocity during infancy, particularly within the first year, followed by progressive deceleration through early childhood [38–40]. The mandible exhibits a similar early growth phase, with peak velocity between 0.4 and 1 year of age, followed by deceleration and a secondary increase during the pubertal growth spurt [38–40]. Among craniofacial structures, the mandible remains the least mature component, retaining the greatest growth potential throughout childhood and adolescence [38–40]. In addition, vertical facial dimensions demonstrate the largest overall increase and continue to develop later than anteroposterior or transverse dimensions [38–40]. The clinical usefulness of sex-specific growth charts is supported by the finding that craniofacial maturation occurs earlier in females than in males at similar ages, which is consistent with previous longitudinal studies [38–40].

## Clinical implications of growth disharmony between oropharyngeal soft tissues and craniofacial structures

The distinct, age-dependent growth patterns of lymphoid tissues and tongue, in relation to craniofacial development, have important clinical implications. As illustrated in Fig. 2, adenoids and palatine tonsils undergo rapid enlargement during early childhood, typically peaking between 5 and 10 years of age, whereas tongue growth is more gradual and region-specific. In contrast, structures such as the pharynx, maxilla, and mandible exhibit delayed and more prolonged growth trajectories. This temporal mismatch between soft tissue expansion and skeletal development results in a transient reduction in airway space [3]. Arens and Marcus described this period — primarily before 8 years of age — as a “window of vulnerability”, during which disproportionate soft tissue volume relative to the craniofacial framework contributes to airway narrowing, particularly in the upper two-thirds of the airway [41]. It should be emphasized that this temporal-mismatch framework represents a conceptual synthesis of the reviewed evidence: although the resulting reduction in airway space is directly observable on imaging, the underlying mechanism is inferred rather than demonstrated by simultaneous longitudinal measurement of soft tissue and skeletal growth. This period coincides with the peak prevalence of pediatric OSA and other related conditions, including otitis media with effusion. Endoscopic evaluation under general anesthesia has demonstrated that airway collapsibility is greatest at the level of the adenoids and palatine tonsils in children with OSA [42].

In healthy children, this imbalance is typically mitigated over time as lymphoid tissues undergo involution while craniofacial structures continue to expand, leading to progressive improvement in airway patency [11, 25, 43]. In contrast, in children with OSA, lymphoid tissue regression may be incomplete or delayed, resulting in persistent airway obstruction extending into later childhood [11, 28, 43]. These differences reflect the dynamic relationship between soft tissue volume and airway growth. As craniofacial structures enlarge, airway dimensions increase, resulting in a progressive reduction in the ratio of lymphoid tissue volume to airway space, even in the presence of persistent lymphoid tissue. Consequently, airway patency is determined not only by absolute tissue size but also by its proportional relationship to the surrounding craniofacial structures [27, 43, 44]. This concept is supported by quantitative imaging studies: maximal adenoid thickness ranges between approximately 10 and 12 mm, [25] reflecting anatomical constraints within the nasopharyngeal space rather than the exaggerated 200% of adult size pattern described in Scammon’s model. Adenoid-to-nasopharynx ratios reach their maximum during early childhood and decline thereafter as airway dimensions increase [10, 43, 45]. Fujioka et al. [26] demonstrated that this ratio peaks at approximately 4.5 years of age — indicating the period of greatest relative airway compromise — after which smaller adenoids regress while larger tissues tend to persist. Computational fluid dynamic modelling has demonstrated that pharyngeal airway shape critically determines internal pressure distribution and regional airway collapsibility in children with OSA, underscoring why proportional tissue-to-airway ratios carry greater functional significance than absolute tissue size alone.

Clinically, these growth dynamics have direct implications for patient assessment and management. Evaluation of airway risk should incorporate both qualitative grading systems — such as the Brodsky tonsil scale and Friedman Tongue Position — and quantitative imaging-based indices reflecting the proportional relationship between soft tissue and airway dimensions. These indices show significant correlations with apnea–hypopnea index (AHI), OSA severity, and oxygen desaturation [44, 46–49]. Observation may be appropriate in selected patients when spontaneous lymphoid involution is anticipated during the transition from early to late childhood. However, because adenotonsillar tissue enlargement peaks between approximately 6 and 10 years of age, this observational period often coincides with critical stages of neurocognitive and physical development. Untreated pediatric OSA during this period has been associated with adverse outcomes, including neurocognitive impairment, behavioral disturbances, metabolic dysfunction, cardiovascular complications, and impaired somatic growth, some of which may persist into adulthood [50–53].

Therefore, clinical decision-making requires careful consideration of both the natural history of lymphoid tissue growth and the potential long-term consequences of untreated airway obstruction. Early identification, risk stratification, and timely intervention are essential, particularly in symptomatic children or those with persistent adenotonsillar hypertrophy [54]. This is clinically significant given that lymphoid tissue hypertrophy is highly prevalent in paediatric orthodontic populations, with one large cephalometric study reporting some form of adenotonsillar hypertrophy in over 90% of subjects (combined adenotonsillar hypertrophy 49.5%, isolated tonsillar hypertrophy 36.6%, isolated adenoid hypertrophy 6.2%) [55].While Scammon’s growth curves provide a useful conceptual framework for understanding generalized lymphoid tissue maturation, they do not fully account for site-specific growth characteristics or the dynamic interaction between soft tissue volume and airway development. Accordingly, clinical evaluation should integrate both absolute and relative measures of tissue size within the context of ongoing craniofacial growth. Importantly, the trajectories described in Table 1 represent population-level tendencies derived largely from single-population cohorts, and considerable individual variability exists in both timing and magnitude of oropharyngeal soft tissue growth (Table 2; Fig. 3B). Notably, obesity appears to alter not just tissue size but the relationship between tissue size and functional severity: adenoid and tonsil size correlate significantly with apnea–hypopnea index (AHI) in normal-weight children but not in obese children, and a smaller magnitude of adenotonsillar hypertrophy is sufficient to produce an equivalent AHI in obese compared with non-obese children [4, 49]. This suggests that imaging- or exam-based tissue size alone is a less reliable indicator of airway risk in obese children, and that clinical evaluation in this population should weight functional and polysomnographic findings more heavily than absolute tissue dimensions. In contrast to skeletal craniofacial maturation, which occurs earlier in females, sex does not appear to significantly influence adenoid or palatine tonsil size in multiple independent cohorts, [27, 30] indicating that soft tissue and skeletal sex-based timing are dissociated processes requiring separate consideration in clinical assessment. Ethnic and population-specific differences in normative reference values further limit direct extrapolation of the ranges in Table 1 across populations, [30] underscoring the need for population-adjusted or anthropometrically normalized reference standards rather than fixed chronological-age cutoffs. Taken together, these findings support a framework in which chronological age provides a population-level starting point, but individualized assessment incorporating weight status, symptom burden, and, where available, population-specific norms is required for accurate airway risk stratification.

Figure 3A illustrates this at the level of raw measurements rather than population percentiles: longitudinal cephalometric data from the same cohort show that adenoid and palatine tonsil area do not decline in a simple monotonic fashion through childhood, but remain statistically indistinguishable across most consecutive age comparisons, with a significant reduction detectable only when early childhood is compared against young adulthood. This contrasts with the ratio-based decline typically emphasized in the literature (e.g., Ad/Np or Tn/Op ratios), which reflects growth of the surrounding airway more than shrinkage of the lymphoid tissue itself — underscoring that the metric chosen to describe “growth” materially changes the resulting curve.

## Summary of key growth patterns of oropharyngeal tissues

**Table 1:** Comparative summary of age-specific growth trajectories of oropharyngeal soft tissues, including the adenoids, palatine tonsils, lingual tonsils, and tongue, with reference to clinical behavior and persistence in the context of sleep-disordered breathing

Tissue	Age of peak growth	Age of maximum size	% Adult size at Peak	Age of regression	Notes on persistence / function
Adenoids	3–5 years (linear growth phase; Arens et al., 2002; Vogler & Pilgram, 2000)	6–8 years (Papaioannou et al., 2013; Jaw et al., 1999); ANR peaks ~ 4.5 yrs (Fujioka et al., 1979)	~ 125% (Ishida et al., 2018)	Begins late childhood; gradual regression by adolescence	Enlargement persists in SDB patients (Papaioannou et al., 2013). Size does not correlate with age in children with SDB. Depth peaks earliest (~ 4 yrs); height and thickness peak at 7–8 yrs.
Palatine Tonsils	3–4 years (rapid early volume expansion; Hosokawa et al., 2020)	8–10 years (Papaioannou et al., 2013; Ishida et al., 2018)	~ 145% (Ishida et al., 2018)	Gradual involution in adolescence; variable in adults	Correlate with age, height, weight, and BMI in healthy children. No age correlation in children with SDB. Oropharyngeal airway remains narrow until ~ 8 yrs, then expands.
Lingual Tonsils	No normative growth data in healthy children	15–18 years; present in 0% healthy children, in 62% of obese children (Gumaraes et al., 2007); and in 33% of post-adenotonsillectomy OSA cases (Fricke et al., 2006); mean diameter 9.5 ± 4.6 mm	Not established	No regression data; not visible on MRI in asymptomatic children	Not a predictable component of normal lymphoid development. Enlargement is compensatory (following adenotonsillectomy) or condition-dependent (OSA, Down syndrome, obesity). Clinical suspicion warranted in persistent OSA.
Tongue	In utero; continuous growth through adolescence	Anterior 2/3: ~8–10 years (~ 75% adult volume by 6 yrs; Temple et al., 2002). Posterior 1/3: full adult size by late adolescence (15–16 yrs)	No overshoot; no involution	No involution; posterior weight increases beyond puberty	General-type growth pattern; no lymphoid-type overshoot. Growth parallels head circumference (Siebert, 1985). Tongue displacement toward oropharynx at age 6–9 yrs: transient reduction in retroglossal airway. Tongue strength increases rapidly from 3–6.5 yrs. Posterior tongue base forms anterior oropharyngeal wall.

Note: “% Adult Size at Peak” values reflect heterogeneous measurement approaches across the cited studies (volumetric MRI/CBCT for adenoids and palatine tonsils; not established for lingual tonsils; linear/area-based for tongue) and should be interpreted as approximations rather than directly comparable, standardized metrics. Normative values are also derived predominantly from single-population cohorts (notably Japanese cephalometric datasets for adenoid/tonsil norms) and may not generalize across ethnic populations [27, 30, 43]

**Table 2:** Individual factors influencing oropharyngeal soft tissue growth and their reported clinical significance

Factor	Adenoids	Palatine tonsils	Lingual tonsils	Clinical significance
Sex	No significant effect on size in longitudinal cephalometric cohorts [27]; however, a large cephalometric cohort (*n* = 404) found significant age-by-sex differences in adenoid width and adenoid/nasopharyngeal ratio, with younger boys showing the largest values [55]	No significant sex correlation in a sonographic normative study, *n* = 274 [30]	Not established	Contrasts with the craniofacial skeleton, where female maturation precedes male at equivalent age (see Craniofacial Growth section); soft tissue and skeletal sex-based timing are dissociated. Sex effects on adenoid size appear inconsistent across cohorts and measurement modalities (ultrasound volume vs. cephalometric linear ratio), suggesting sex-based variability may be population- or method-dependent rather than absent [55]
Obesity / BMI	Independently increases OSA risk (OR 1.89, 95% CI 1.19–3.00); size correlates with AHI in normal-weight but not obese children [11, 46]	Independently increases OSA risk (OR 2.89–3.15); smaller hypertrophy produces an equivalent AHI in obese vs. non-obese children [4, 11, 49]	Present in 62% of obese children vs. 0% of healthy controls [31]	Tissue size on imaging/exam is a less reliable severity marker in obese children; functional and polysomnographic findings should be weighted more heavily
Height / weight (non-obese range)	Correlates with anthropometrics in some cohorts	Correlates with age, height, weight, and BMI; height more influential in early childhood, weight later [30]	Not established	Supports proportional, anthropometrically adjusted rather than fixed chronological-age reference standards
Ethnicity / population	Normative datasets are largely population-specific (e.g., Japanese cephalometric cohorts) [10, 27, 43]; a Singapore-Chinese cohort (*n* = 404) showed a modestly higher adenoid/nasopharyngeal ratio (0.48–0.55) than a predominantly White American cohort (0.45), with smaller minimum posterior airway space [55]	Population-specific reference differences reported across cohorts [30]	Not established	Ranges in Table 1 should not be applied cross-population without validation; direct population comparisons confirm meaningful differences in adenoid proportionality and airway space between Asian and White cohorts [55]
Allergic/inflammatory disease, recurrent infection	Associated with sustained or delayed involution (qualitative evidence) [23, 56]	Associated with recurrent hypertrophy	Compensatory enlargement following adenotonsillectomy or in persistent OSA [32]	“Age of regression” in Table 1 reflects a population median, not a guaranteed individual endpoint
Syndromic conditions (e.g., Down syndrome)	Not systematically studied	Not systematically studied	Present in 50% of Down syndrome + OSA cases vs. 33% of general persistent OSA cases [32–34]	Syndromic children warrant a lower threshold for imaging-based reassessment regardless of chronological age

## Conclusion

Oropharyngeal soft tissue growth is neither uniform nor fully captured by Scammon’s classical lymphoid growth curve. Unlike the thymus, the pharyngeal lymphoid tissues reach maximal size at a younger age and with less pronounced overshoot than the classical 200% of adult size pattern suggests. The adenoids typically peak between 5 and 8 years; palatine tonsils reach maximum dimensions between 8 and 10 years; and lingual tonsils currently lack clearly established normative developmental trajectories in healthy children. The absence of standardized longitudinal or cross-sectional data in asymptomatic populations represents a significant gap in the literature, and any characterization of their ‘normal’ developmental pattern would be premature based on currently available evidence. Lingual tonsil hypertrophy instead appears to represent a compensatory or condition-dependent phenomenon rather than a normal growth pattern [32].

Crucially, current evidence distinguishes between normal growth patterns — which may involve a peak in lymphoid tissue size around age 7–10 before steady decline — and the pathological enlargement seen in children with OSA or persistent airway obstruction, where tonsillar and adenoid size is often maintained and disproportionate to the airway lumen. The tongue follows a general-type growth pattern with distinct regional timing, and its posterior third continues to enlarge into adolescence, contributing to pharyngeal airway dynamics in ways that are not predicted by classical models.

An integrative, structure-specific clinical framework — one that accounts for the proportional relationship between each oropharyngeal tissue and the surrounding developing craniofacial architecture — is essential for informed orthodontic assessment, airway risk stratification, and timely intervention in children with or at risk for sleep-disordered breathing. Recognition of the “window of vulnerability” between approximately 5 and 10 years underscores the urgency of early clinical evaluation in symptomatic patients.

## Data Availability

Data sharing is not applicable to this article as no datasets were generated or analyzed during the current study.
